# P-100. What is a Postoperative Spine Infection?

**DOI:** 10.1093/ofid/ofaf695.329

**Published:** 2026-01-11

**Authors:** Seyed Mohammad Amin Alavi, Fabio Borgonovo, Francesco Petri, Takahiro Matsuo, Andrea Gori, Jeremy D Shaw, Aaron J Tande, Elie F Berbari

**Affiliations:** Ahvaz Jundishapur University of Medical Sciences, Ahvaz, Khuzestan, Iran; Mayo Clinic, Rochester, Minnesota; Mayo Clinic, Rochester, Minnesota, Rochester, Minnesota; Mayo Clinic, Rochester, Minnesota; Department of Infectious Diseases, “Luigi Sacco” University Hospital, ASST FBF Sacco, 20157 Milan, Italy., Milan, Lombardia, Italy; Intermountain Neurosciences, Intermountain Health, Salt Lake City, UT, USA, Salt Lake, Utah; Division of Public Health, Infectious Diseases and Occupational Medicine, Department of Medicine, Mayo Clinic College of Medicine and Science, Mayo Clinic, Rochester, 55905, MN, USA., Rochester, Minnesota; Mayo Clinic, Rochester, Minnesota

## Abstract

**Background:**

The increased frequency of spinal surgeries has led to a rise in postoperative spine infections (PSIs), now recognized as the third most common surgical complication. Currently, there is no standardized definition of PSI, complicating clinical management and research. This meta-epidemiological study aimed to develop clear diagnostic criteria to assist clinicians and researchers in identifying and reporting PSIs consistently.

**Figure:**
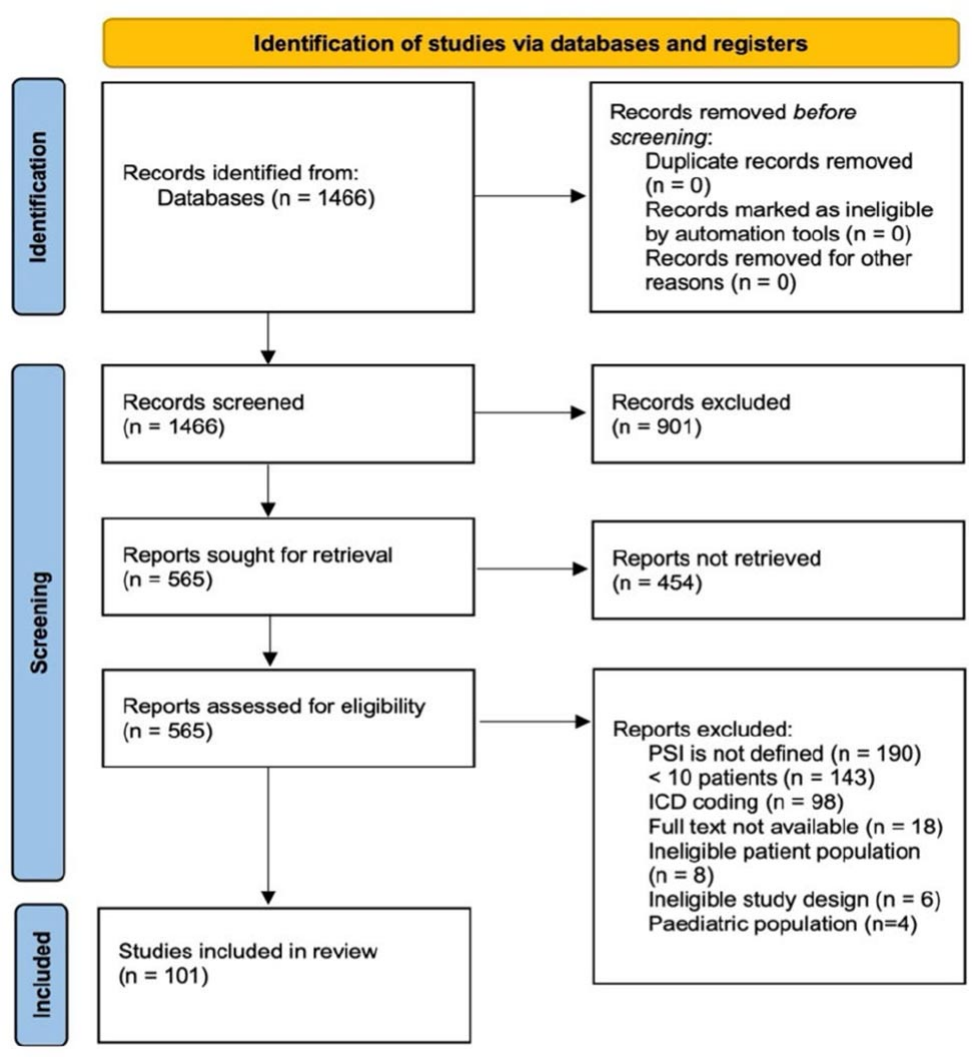
2020 PRISMA flow diagram Sankey diagram showing the distribution of combination of criteria or definitions used by the authors. This refers to 21 articles that used a definition derived by the authors.

**Figure:**
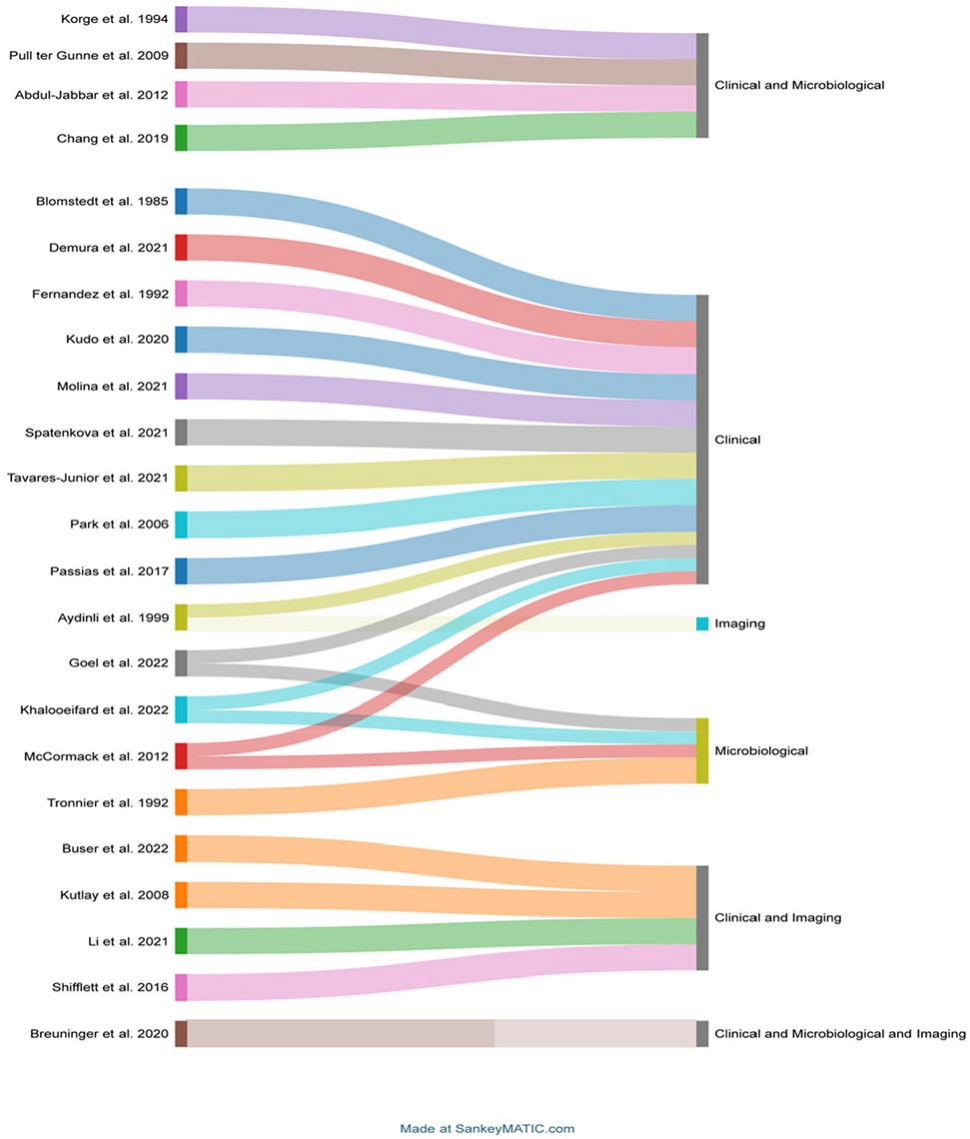

**Methods:**

Following PRISMA guidelines, a comprehensive literature search was conducted by a medical librarian using Ovid MEDLINE (March 14, 2024). Studies were included if they reported on at least ten adult patients diagnosed with PSI and provided clear diagnostic definitions. The primary objective was to evaluate existing PSI diagnostic criteria and categorize them into thematic clusters. We constructed standardized diagnostic combinations using 13 predefined criteria derived from clinical practice guidelines and prior research (Table 1).

**Figure:**
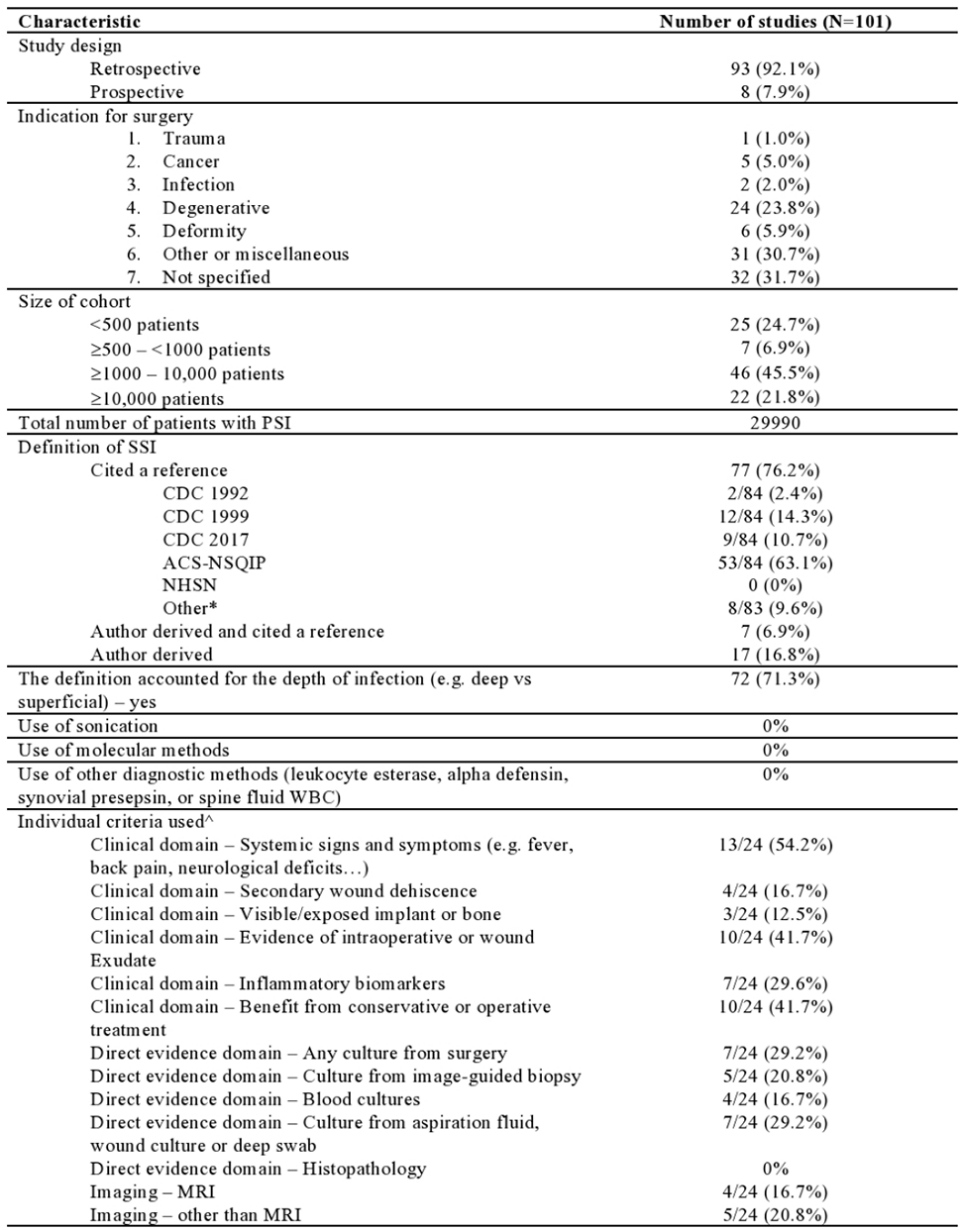
Characteristics of the included studies *Total can be more than 100% since some variables can be present in multiple categories. This refers to 21 articles that used a definition derived by the authors. For articles #411 and #630 categorization was not possible as the definition provided only information about the depth of infection and not the criteria for diagnosis. Article #1148 was included as, defining “presumed aspetic” cases meant, by definition, also defining infectious cases. ***Abbreviations.*** The American College of Surgeons-National Surgical Quality Improvement Program (ACS-NSQIP), Centers for Disease Control and Prevention (CDC), The National Healthcare Safety Network (NHSN), Preferred Reporting Items for Systematic Review and Meta-analysis (PRISMA), Magnetic Resonance Imaging (MRI)

**Results:**

From an initial 1,466 studies screened, 101 were included (Figure 1). Most studies were retrospective (91/101, 92.1%), and a majority (77/101, 76.2%) relied on established criteria such as those from the American College of Surgeons-National Surgical Quality Improvement Program (ACS-NSQIP) or the CDC. Seven studies combined predefined criteria with additional detailed explanations. Among the remaining studies using independent definitions, clinical signs and symptoms were the most common criteria (13/24, 54.2%) (Table 1). Criteria were grouped into clinical, microbiological, and radiological categories, with most studies primarily using clinical criteria alone or in combination with microbiological or imaging findings (Figure 2).

**Conclusion:**

There was no clear consensus in the literature on what defines a PSI. Our findings emphasize the necessity of establishing a standardized PSI definition that integrates clinical, microbiological, and radiological criteria. Implementing such a unified framework could enhance diagnostic accuracy, address existing limitations associated with hardware complications, wound drainage interpretations, and imaging ambiguities, and improve upon the current shortcomings of surveillance systems.

**Disclosures:**

Jeremy D. Shaw, MD, Globus: Grant/Research Support|Purgo Scientific: Advisor/Consultant|Purgo Scientific: Stocks/Bonds (Private Company)

